# Influence of 20–Year Organic and Inorganic Fertilization on Organic Carbon Accumulation and Microbial Community Structure of Aggregates in an Intensively Cultivated Sandy Loam Soil

**DOI:** 10.1371/journal.pone.0092733

**Published:** 2014-03-25

**Authors:** Huanjun Zhang, Weixin Ding, Xinhua He, Hongyan Yu, Jianling Fan, Deyan Liu

**Affiliations:** 1 State Key Laboratory of Soil and Sustainable Agriculture, Institute of Soil Science, Chinese Academy of Sciences, Nanjing, China; 2 Forests NSW, NSW Department of Primary Industries, West Pennant Hills, New South Wales, Australia; 3 School of Plant Biology, University of Western Australia, Crawley, Western Australia, Australia; Tennessee State University, United States of America

## Abstract

To evaluate the long–term effect of compost (CM) and inorganic fertilizer (NPK) application on microbial community structure and organic carbon (OC) accumulation at aggregate scale, soils from plots amended with CM, NPK and no fertilizer (control) for 20 years (1989–2009) were collected. Soil was separated into large macroaggregate (>2,000 μm), small macroaggregate (250–2,000 μm), microaggregate (53–250 μm), silt (2–53 μm) and clay fraction (<2 μm) by wet-sieving, and their OC concentration and phospholipid fatty acids (PLFA) were measured. The 20-year application of compost significantly (*P*<0.05) increased OC by 123–134% and accelerated the formation of macroaggregates, but decreased soil oxygen diffusion coefficient. NPK mainly increased OC in macroaggregates and displayed weaker influence on aggregation. Bacteria distributed in all aggregates, while fungi and actinobacteria were mainly in macroaggregates and microaggregates. The ratio of monounsaturated to branched (M/B) PLFAs, as an indicator for the ratio of aerobic to anaerobic microorganisms, increased inversely with aggregate size. Both NPK and especially CM significantly (*P*<0.05) decreased M/B ratios in all aggregates except the silt fraction compared with the control. The increased organic C in aggregates significantly (*P*<0.05) negatively correlated with M/B ratios under CM and NPK. Our study suggested that more efficient OC accumulations in aggregates under CM–treated than under NPK–treated soil was not only due to a more effective decrease of actinobacteria, but also a decrease of monounsaturated PLFAs and an increase of branched PLFAs. Aggregations under CM appear to alter micro-habitats to those more suitable for anaerobes, which in turn boosts OC accumulation.

## Introduction

Sequestration of carbon (C) in soils has gained increasing recognition in recent years for its role in global environmental change [1]. Soil aggregation affects soil properties such as aeration and water infiltration and alters micro–habitats and activities of microorganisms [2]. A growing body of work has revealed a close relationship between aggregation and soil organic C (OC) concentrations [3], [4] as OC stabilization in aggregates is the principal mechanism controlling OC turnover and sequestration [5], [6], [7].

The potential of soils to sequester C depends on soil type, management practices, and soil aggregate structure [5], [8], [9]. Previous studies have suggested that OC is more effectively protected and more stable in microaggregates, whereas macroaggregates provide a niche for the storage of labile C [3], [5], [10]. Fertilization, a common agricultural practice, has been found to significantly influence aggregate formation and OC distribution in aggregates [11], [12]. For example, increases in OC following organic manure application were observed in aggregates of all sizes, especially in macroaggregates [11], [13]. Kong *et al*. [14] found that the majority of OC derived from organic material was preferentially sequestered in microaggregates within small macroaggregates. In contrast, Sarkar *et al.* [15] and Bhattacharyya *et al.* [16] found that the highest rate of increase in OC following repeated inorganic fertilizer applications primarily occurred in the silt and clay fractions of OC–poor soils.

The turnover of OC in soil aggregates is not only determined by the physical protection offered by that fraction, but also by the abundance and community of microorganisms, especially Gram–positive (G^+^) bacteria and actinobacteria [17], [18], [19]. Aggregates provide spatially heterogeneous habitats for microorganisms characterized by different substrates, oxygen concentrations and water contents [20]. In turn, microorganisms provide further ecosystem functions through their effects on soil structure by binding soil particles and organic matter to create aggregates [9], [21]. Shifts in microbial community and function in response to different agricultural management practices can markedly alter rates of organic C loss from soils. Determining how fertilization affect the distribution of microbial functional groups among aggregates can lead to a better understanding of the differences in soil OC turnover under different management practices [2], [22]. A previous study showed that different fertilizer treatments such as nitrogen fertilizer, green manure and sewage sludge did not dramatically alter the distribution pattern of bacteria in aggregates <200 μm [20].

The abundance and community structure of microorganisms are very responsive and can provide immediate and precise information on changes occurring in soil. In contrast, OC concentrations in soil and aggregates change at a slower rate [23]. To date, limited information has been available on the relationship between OC turnover and microbial community structure in soil aggregates. A long–term field experiment has been established in the Northern China plain to monitor changes in soil organic C under organic and inorganic fertilizer applications. In the present study, the abundance and community structure of microorganisms in aggregates were measured. The objectives of this study were to: (1) identify how repeat inorganic and organic fertilizer applications affect the distribution and community structure of microorganisms in aggregates; and (2) evaluate the relationship between OC accumulation and microbial community structure in aggregates.

## Materials and Methods

### Field Experiment and Soil Sampling

A long–term field experiment was set up in September 1989 to monitor the dynamic variation in OC following applications of compost and inorganic fertilizer to an intensively cultivated fluvo–aquic soil at the Fengqiu Agro–ecological Experimental Station, Chinese Academy of Sciences, Henan Province, China (35°00′N, 114°24′E). The soil was developed from alluvial sediments of the Yellow River and classified as Aquept [24]. Prior to the experiment, the field had been cultivated under a similar agricultural regime for at least 50 years, so the heterogeneity of soil fertility was minimal. The characteristics of the surface soil (0–20 cm) in September 1989 were listed in Table 1.

**Table 1:** Characteristics of the soil sampled in September 1989.

Texture (Sand, Silt, Clay)	pH_H2O_	Organic C	Total N	Total P	Total K	Inorganic N
(%)	(extract 1∶5, w/v)	(g C kg^–1^)	(g N kg^–1^)	(g P kg^–1^)	(g K kg^–1^)	(mg N kg^–1^)
52,33,15	8.65	4.48	0.43	0.5	18.6	9.51

The experiment was based on a random design including three fertilization treatments with four replicate plots (9.5 m×5 m each): (1) no–fertilization control (control), (2) inorganic fertilizer NPK (NPK) and (3) compost (CM). Winter wheat (*Triticum aestivum*) was annually rotated with summer maize (*Zea mays*). A total of 150 kg N ha^–1^ for each crop was applied in the NPK and CM treatments. In the NPK treatment, urea was used at 60 or 90 kg N ha^–1^ as basal fertilizer in early June or early October for maize or wheat, respectively, and at 90 or 60 kg N ha^–1^ as supplement fertilizer in late July or late February for maize or wheat, respectively. In the NPK treatment, 75 kg P_2_O_5_ ha^–1^ (calcium superphosphate) and 150 kg K_2_O ha^–1^ (potassium sulfate) were also added to each crop. In the CM treatment, a total of 2.76 tons ha^–1^ compost (made from wheat straw, oil cake and cotton cake, with a C/N ratio of approximately 8), which contained 1,164 kg C, 150 kg N, 51 kg P_2_O_5_ and 65 kg K_2_O, was applied as basal fertilizer in early June for maize and early October for wheat. To match the same amounts of P and K with the NPK treatment, 24 kg P_2_O_5_ ha^–1^ (calcium superphosphate) and 85 kg K_2_O ha^–1^ (potassium sulfate) were added to the compost prior to the application. No fertilizer was applied in the control treatment. Fertilizers and composts were broadcasted onto the soil surface by hand and the surface soil (0–20 cm) was immediately tilled. Seeds of 195 kg wheat ha^–1^ and 37.5 kg maize ha^–1^ were immediately sown into the soil by hand after the basal fertilization. Seeding rows were 70 cm wide for maize and 15 cm wide for wheat cultivation. Over the entire 20–year period, field management followed local practices.

Ten soil cores (0–20 cm) from each replicate plot were collected with a 2.5 cm diameter auger and then mixed to form one composite sample on 7 June 2009 after wheat harvest. Fresh samples were stored at 4°C and immediately transported to the laboratory for wet–sieving and phospholipid fatty acids (PLFA) analyses, while air–dried and sieved soils were used to determine the OC content by the wet oxidation–redox titration method [25]. A further eight undisturbed cylinder soil samples (100 cm^3^) were taken from each plot to establish the soil water retention curve and bulk density.

### Soil Water Retention Curve and the Effective Diffusion Coefficient of Oxygen

Water retention curves were determined with a ceramic pressure plate assembly at equilibrium matric potentials of −0.1, −0.2, −1, −3.5, −6, −10, −33, −50, −100, −200, −500 and −1,500 kPa in a pressure chamber. The obtained data were used to calculate the soil water retention curves and to derive the van Genuchten parameters by RETC (RETention Curve) software [26] using the following equation:
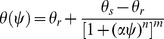
where *θ* is the soil water content (cm^3^ cm^−3^), *θ_s_* and *θ_r_* are the saturated and residual water contents (cm^3^ cm^−3^), respectively, *ψ* is the matric potential (kPa) as indicated by pressure head, and the parameters of *α*, *n* and *m* (*m* = 1*–*1*/n*) are dimensionless.

According to the capillary rise theory, the pore size can be calculated using the following equation when the soil is hydrophilic at 20°C of water [27]:
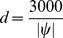
where *d* is the diameter of pore (μm), and |*ψ*| is the absolute matric potential (kPa).

The effective diffusion coefficient (*D*) of oxygen through the pore space of the soil (m^2^ s^–1^) was calculated as follows [28]:
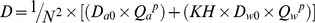
where *N* is soil porosity, *D_a0_* is the free diffusion coefficient in air (1.8×10^–5^ m^2^ s^–1^ at 20°C), *D_w0_* is the free diffusion coefficient in water (2.2×10^–9^ m^2^ s^–1^ at 20°C), *Q_a_* and *Q_w_* are the proportion of soil porosity occupied by air and water, respectively, i.e. *Q_a_*+*Q_w_* = 1, and *p* is a power constant (*p* = 3.4).

*Q_w_* was calculated as follows:
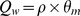
where *ρ* is the soil bulk density, and *θ_m_* is the soil moisture content.

### Soil Fractionation

The water–stable aggregates of moist soils were separated using the wet–sieving protocol [29]. One hundred grams of moist soil samples (on an oven–dried basis) were submerged in deionized water for 5 min at room temperature on top of a 2,000–μm sieve. The sieve was manually moved up and down 3 cm, 50 times over a 2–min period. The fraction remaining on the 2,000–μm sieve was collected in a pre–weighed aluminum pan. Water plus the filtered soil was poured through a 250–μm sieve, and the sieving procedure repeated. Water plus the <250 μm fraction of soil was poured through a 53–μm sieve, and the sieving procedure repeated. To separate the silt (2–53 μm) from the clay (<2 μm) fraction, the remaining suspension was poured into centrifuge bottles and centrifuged at approximately 1,000 rpm for 3 min at 15°C. To obtain the clay fraction, all supernatants collected in several centrifuge bottles were centrifuged at 4,500 rpm for 30 min at 15°C. Large macroaggregate (>2,000 μm), small macroaggregate (250–2,000 μm), microaggregate (53–250 μm), silt (2–53 μm) and clay (<2 μm) fractions were obtained from the tested soils. An aliquot of each fraction was used to determine moisture content. Another subsample of each fraction was dried at 50°C to determine the OC content. The remaining sample was used to measure the microbial biomass and community structure using PLFA method.

### PLFA Measurement

PLFAs were extracted using a modified Bligh–Dyer technique as described by Brant *et al.* [30]. In brief, three grams of soil sample (on an oven–dried basis) were incubated in a 2∶1:0.8 solution of methanol, chloroform, and phosphate buffer. Soil extracts were centrifuged and the chloroform phases collected. Phospholipids were separated from glycolipids and neutral lipids using silicic acid bonded solid–phase–extraction columns. The phospholipid fractions were dried under a stream of nitrogen at 37°C. The fatty acid methyl esters (FAME) were produced through mild alkaline methanolysis, and the FAME were dried under nitrogen at 37°C. Finally, the FAME were dissolved in hexane, which contained a 19∶0 (methyl nonadecanoate fatty acid) FAME standard. Samples were analyzed on a Shimadzu GC–MS QP 2010 PLUS (Shimadzu, Kyoto, Japan). Peaks were identified based on comparing retention times with known standards. Concentrations of each PLFA were obtained by comparing peak areas with the 19∶0 FAME standards.

PLFAs have been used as biomarkers for various groups of microorganisms [31]. The PLFAs 16∶3ω3, 18∶1ω9, 18∶2ω6,9 18:2ω9,12 and 20∶1ω9 are used as biomarkers for fungi [19], [32]; 16∶1ω5 is generally attributed to arbuscular mycorrhizal fungi [33]; 10Me17∶0, 10Me18∶0, and 10Me20∶0 for actinobacteria [34], [35]; 3OH–15∶0, 14∶0, 15∶0, 16∶0, 17∶0, 18∶0, 19∶0 and 20∶0 for general bacteria; a15∶0, i15∶0, a16∶0, i16∶0, a17∶0, i17∶0 and i19∶0 for G^+^ bacteria [34]; and cy17∶0, cy19∶0, 16∶1ω7c, 16∶1ω7t, 18∶1ω7c and 18∶1ω7t for Gram–negative (G^–^) bacteria [34], [35]. According to Bossio *et al.* [36], 16∶1ω7c, 16∶1ω7t, 18∶1ω7c, 18∶1ω7t, 18∶1ω9 and 16∶1ω5 are monounsaturated PLFAs, while 3OH–15∶0, a15∶0, i15∶0, a16∶0, i16∶0, a17∶0, i17∶0, i19∶0, 10Me17∶0, 10Me18∶0 and 10Me20∶0 are branched PLFAs. The ratio of monounsaturated to branched PLFAs is regarded as an indicator for the ratio of aerobic to anaerobic microorganisms and the development of aerobic conditions [36], [37].

### Statistical Analysis

The recovery rate of microbial PLFAs in all aggregates was calculated as follows:
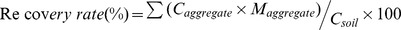
where *C*_aggregate_ is the concentration of microbial PLFAs in aggregates (nmol g^–1^ aggregate), *M*_aggregate_ is the proportion of aggregates in whole soil by mass (kg kg^–1^ soil), and *C*_soil_ is the concentration of microbial PLFAs in soil (nmol g^–1^ soil).

Statistically significant differences among the treatments were determined using analysis of variance (one–way ANOVA) and Least Significant Difference (LSD) calculations at the 5% level with SPSS 18 for windows. Redundancy analysis (RDA) and regression analyses were used to test relationships between the increased organic C concentration in the CM and NPK treatments compared with the control and the abundance of microorganisms in aggregates in the CM and NPK treatments.

## Results

### Aggregate Mass Distribution and Aggregate–associated OC Concentration

**Figure 1:**
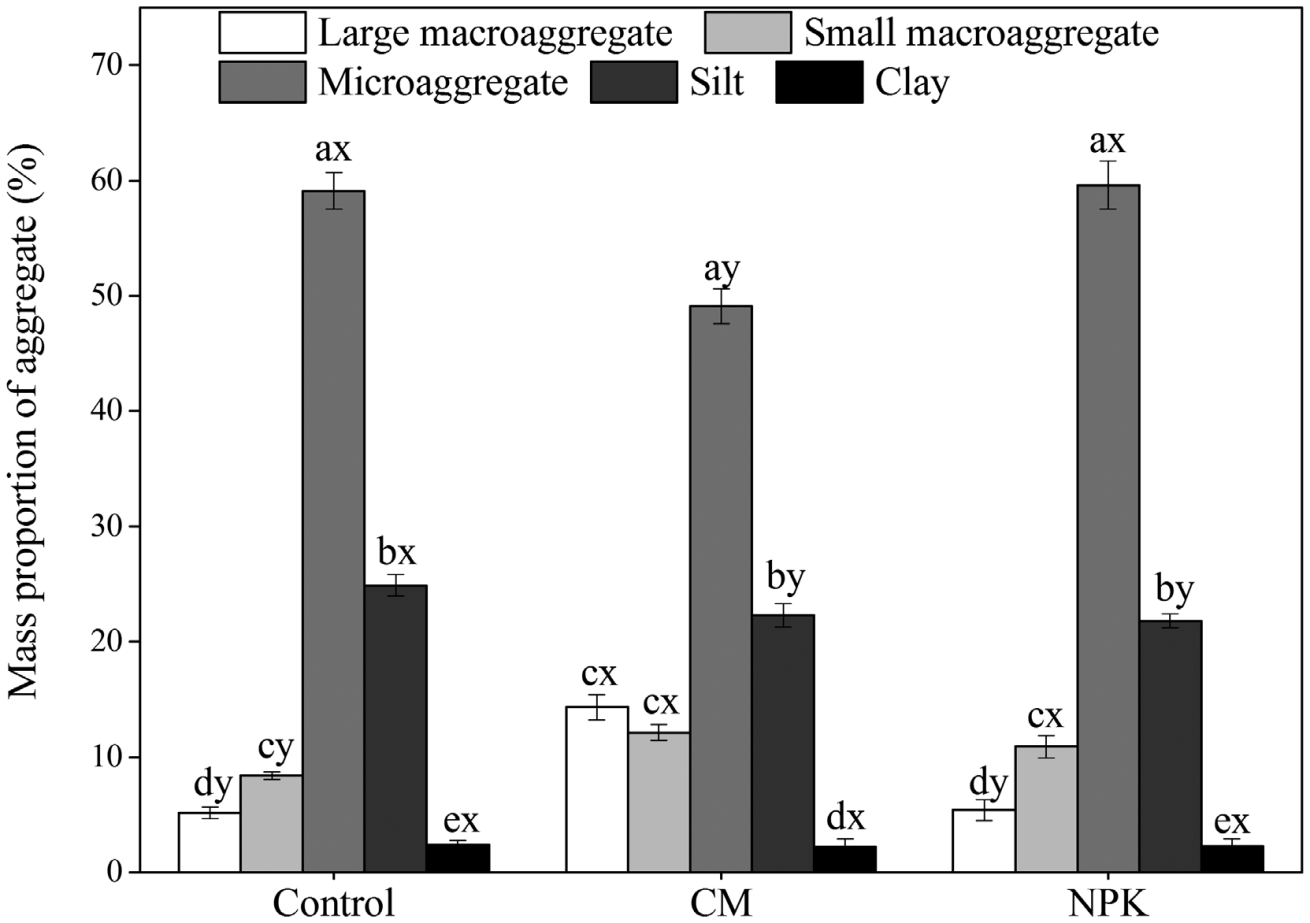
Effect of long–term applications of compost and fertilizer NPK on the mass proportion of aggregates in soil. Vertical bars indicate the standard error of the means (*n* = 4). Different letters denote significant differences between aggregates in the same treatment (a, b, c, d, e) and between treatments with the same aggregate (x, y, z) at *P<*0.05.

Compost applications significantly (*P*<0.05) increased the mass proportion of large and small macroaggregates by 175% and 44%, respectively, at the expense of reduction in the silt fraction or microaggregate compared with the control. Inorganic fertilizer NPK only significantly (*P*<0.05) increased the proportion of small macroaggregates by approximately 30% (Fig. 1). The highest OC concentration was observed in small macroaggregates in all treatments, while the lowest occurred in the clay fraction (Table 2). Compost application more efficiently increased organic C in all aggregates than fertilizer NPK, and significantly (*P*<0.05) increased the values by 123–134% compared with the control. NPK mainly increased organic C in large and small macroaggregates, and to a lesser extent those in microaggregate, silt and clay fractions.

**Table 2:** Effect of long–term applications of compost and fertilizer NPK on organic C concentrations (g C kg^–1^ aggregate) in aggregates.

Treatment	Large macroaggregate (>2,000 μm)		Small macroaggregate(250–2,000 μm)		Microaggregate(53–250 μm)		Silt fraction(2–53 μm)		Clay fraction (<2 μm)	
	Concentration (g C kg^−1^)	Increase (%)	Concentration (g C kg^−1^)	Increase (%)	Concentration (g C kg^−1^)	Increase (%)	Concentration(g C kg^−1^)	Increase (%)	Concentration(g C kg^−1^)	Increase (%)
Control	4.32±0.04 cz	–	8.56±0.06 az	–	4.28±0.07 cz	–	4.57±0.22 bz	–	4.04±0.05 dy	–
CM	9.93±0.09 cx	130	20.01±0.29 ax	134	9.67±0.01 dx	126	10.37±0.06 bx	127	9.02±0.07 ex	123
NPK	8.16±0.10 by	89	17.82±0.11 ay	108	6.01±0.16 dy	40	6.72±0.10 cy	47	5.76±0.08 ey	43

Mean ± standard deviation (n = 4).

Increase (%) = (organic C in fertilization treatments – organic C in the control treatment)/organic C in the control treatment×100.

Different letters denote significant differences between aggregates with the same treatment (a, b, c, d, e) and between treatments with the same aggregate (x, y, z) at *P*<0.05, respectively.

### Pore Size and Effective Diffusion Coefficient of Oxygen

**Figure 2:**
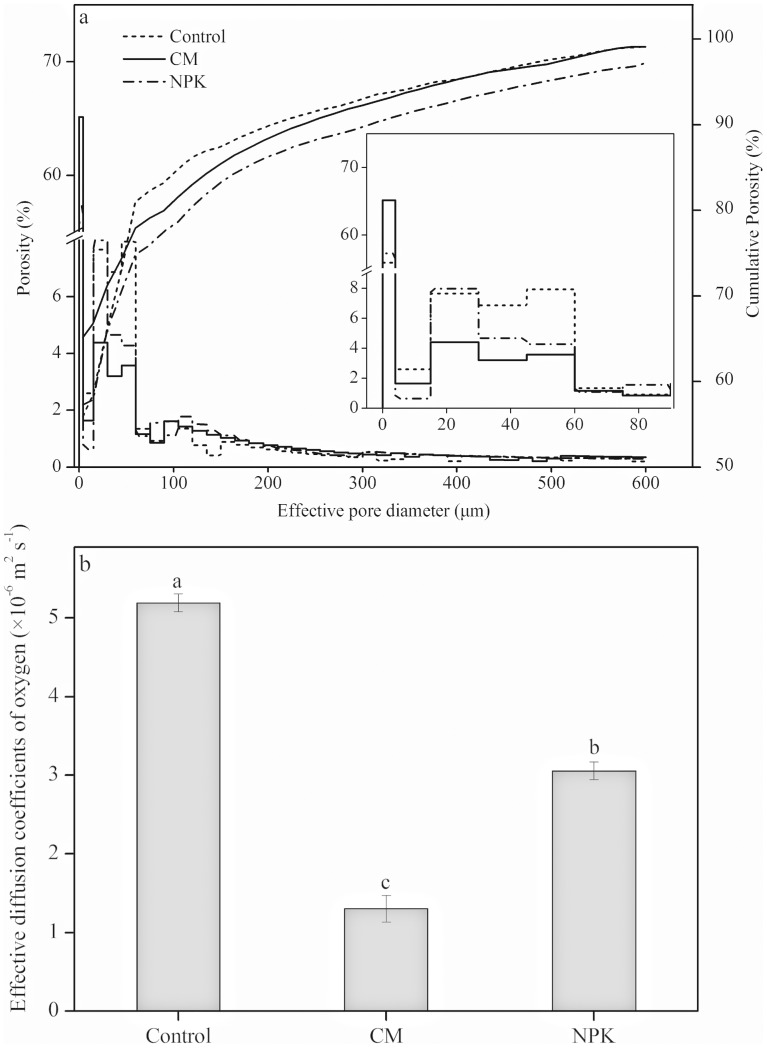
Effect of long–term applications of fertilizer NPK and compost on the proportions of pore volumes of different diameter (a) and effective diffusion coefficient of oxygen (b) in the soil. Vertical bars indicate the standard error of the means (*n* = 4). Different lowercase letters denote significant differences between treatments at *P<*0.05.

The proportion of pores with a neck diameter <4 μm in the CM treatment amounted to 65.12%, which was significantly (*P*<0.05) higher than in the control and NPK treatments, the latter being 55.87 and 57.28%, respectively (Fig. 2a). In contrast, the proportion of pores with a neck diameter of 15–60 μm showed a decrease in the order: control>NPK>CM and there was a significant difference between treatments. Pores greater than 300 μm were significantly (*P*<0.05) increased by fertilization, but there was no obvious difference between CM and NPK treatments. The highest effective diffusion coefficient of oxygen was observed in the control treatment, peaking at 5.19×10^–6^ m^2^ s^–1^, while the lowest occurred in the CM treatment, measuring only 1.30×10^–6^ m^2^ s^–1^ (Fig. 2b).

### Concentrations of Bacterial, Fungal and Actinobacterial PLFAs in Aggregates

**Figure 3:**
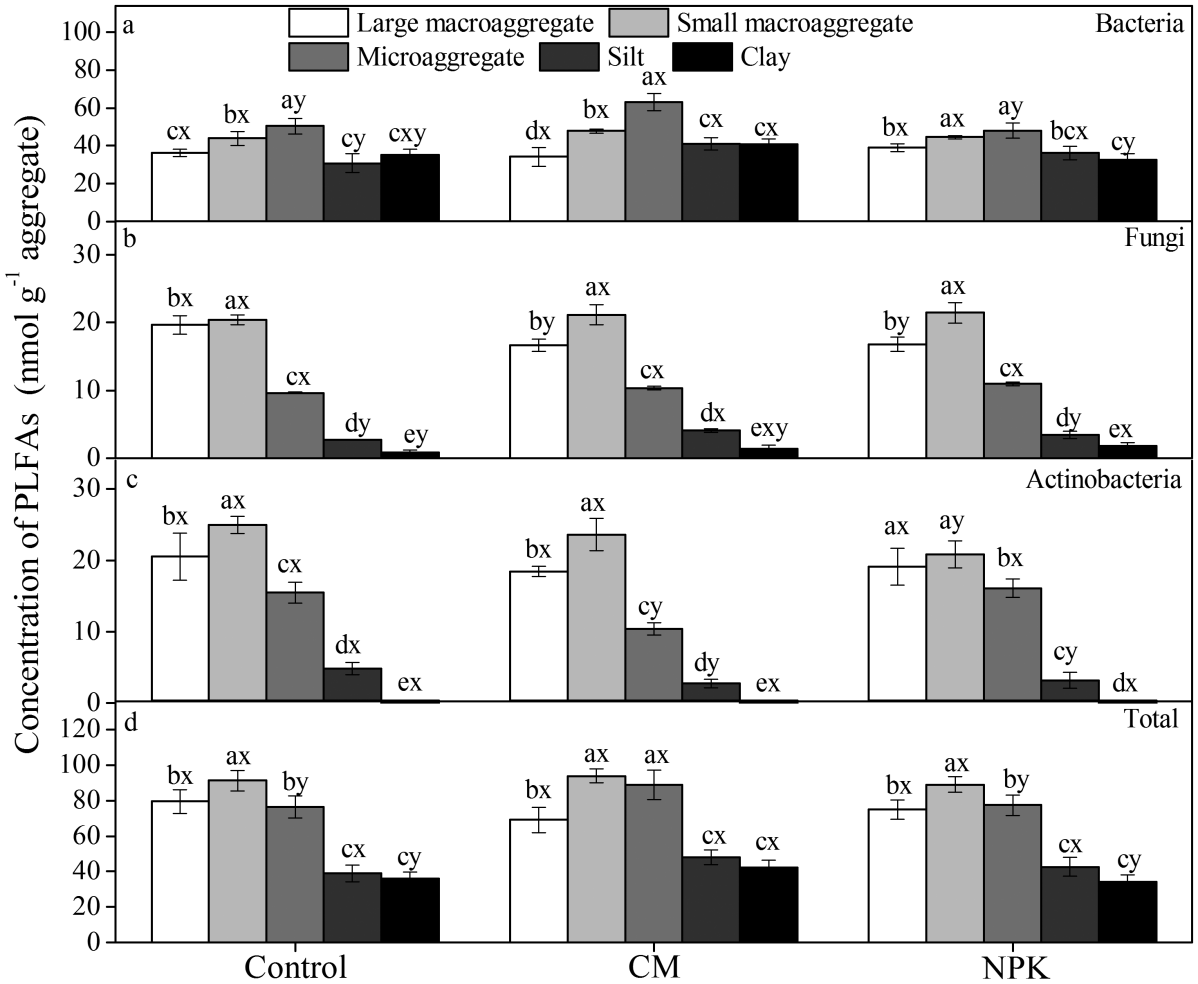
Effect of long–term applications of fertilizer NPK and compost on concentrations (nmol g^–1^ aggregate) of bacterial, fungal, actinobacterial and total PLFA in aggregates. Values are means (*n* = 4) with standard error. Different letters denote significant differences between aggregates with the same treatment (a, b, c, d, e) and between treatments with the same aggregate (x, y, z) at *P*<0.05, respectively.

The recovery rate of microbial PLFAs in all aggregates compared with soil was 95–100%. The highest concentrations of microbial PLFAs were found in small macroaggregates, ranging from 89.00 to 93.93 nmol g^–1^ aggregate, and the lowest was found in the clay fraction for all treatments (Fig. 3d). The NPK application did not significantly (*P*>0.05) affect the concentration of total microbial PLFAs compared with the control. In contrast, compost significantly (*P*<0.05) increased the concentration of total PLFAs in the microaggregates and clay fraction.

The concentrations of bacterial PLFAs in microaggregates in all treatments varied from 47.97 to 63.07 nmol g^–1^ aggregate, being significantly (*P*<0.05) higher than in other aggregates, while the lowest was in the clay fraction (Fig. 3a). Compost significantly (*P*<0.05) increased the concentrations of bacterial PLFAs in microaggregates, silt and clay fractions, but fertilizer NPK only significantly (*P*<0.05) increased those in the silt fraction compared with the control.

The highest concentrations of fungi, varying from 20.39–21.53 nmol g^–1^ aggregate, were observed in the small macroaggregates, followed by large macroaggregates, while the lowest were measured in the clay fraction, being only 0.81–1.83 nmol g^–1^ aggregate in the three treatments. The concentrations of fungi in large macroaggregates were significantly (*P*<0.05) reduced by NPK and compost application, but were markedly increased in the silt fraction after compost was applied (Fig. 3b).

No actinobacterial PLFAs were found in any of the clay fractions (Fig. 3c). The maximum concentration of actinobacteria was observed in small macroaggregates, and the minimum in the silt fraction. The application of compost significantly (*P*<0.05) reduced their concentration in all aggregates, whereas NPK fertilization principally lowered the concentration in macroaggregates and the silt fraction.

### Concentrations of Monounsaturated and Branched PLFAs in Aggregates

**Figure 4:**
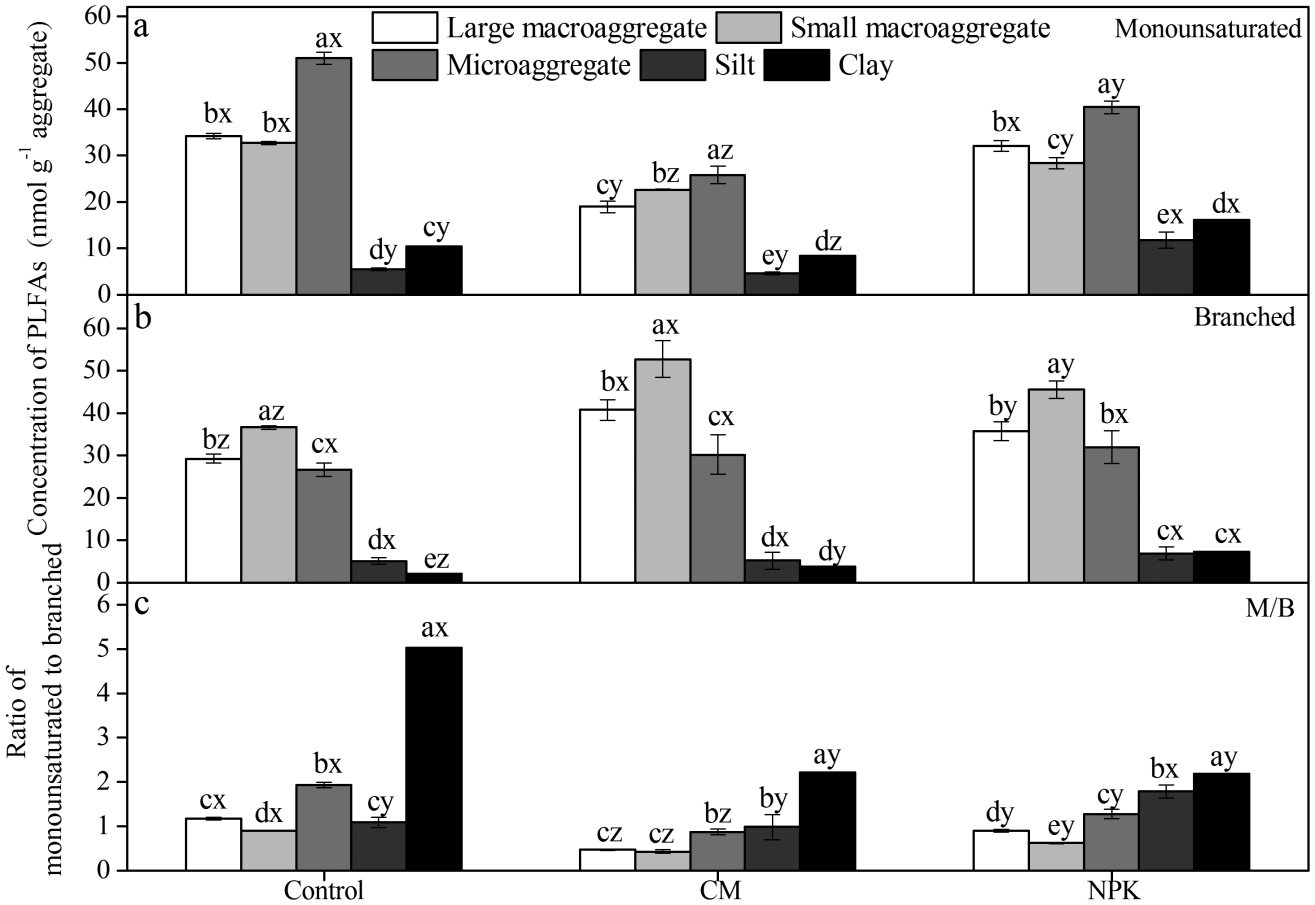
Effect of long–term applications of fertilizer NPK and compost on concentrations (nmol g^–1^ aggregate) of monounsaturated and branched PLFA in aggregates. Values are means (*n = *4) with standard error. Different letters denote significant differences between aggregates with the same treatment (a, b, c, d, e) and between treatments with the same aggregate (x, y, z) at *P*<0.05, respectively.

The concentration of monounsaturated PLFAs, which were mainly composed of G^–^ bacterial PLFAs, was highest in microaggregates and lowest in the silt fraction in all treatments (Fig. 4a). Compost significantly (*P*<0.05) reduced the concentrations of monounsaturated PLFAs in all aggregates compared with the control, while fertilizer NPK reduced their levels in small macroaggregates but increased their concentrations in the silt and clay fractions. In contrast, fertilization, regardless of type, significantly (*P*<0.05) elevated the concentrations of branched PLFAs including G^+^ bacterial and actinobacterial PLFAs in large and small macroaggregates and clay fractions compared with the control (Fig. 4b). However, compost exerted a larger effect in large and small macroaggregates and weaker influences in the clay fraction than NPK. The ratio of monounsaturated to branched PLFAs (M/B ratio) increased as aggregate size reduced in all treatments, except for small macroaggregates and the silt fraction in the control treatment and small macroaggregates in the NPK treatment (Fig. 4c). Compost application reduced M/B ratios more efficiently in all aggregates than NPK, and the latter significantly (*P*<0.05) increased the M/B ratio in the silt fraction.

### Correlation between Microbial PLFAs and the Increase of OC in Aggregates

**Figure 5:**
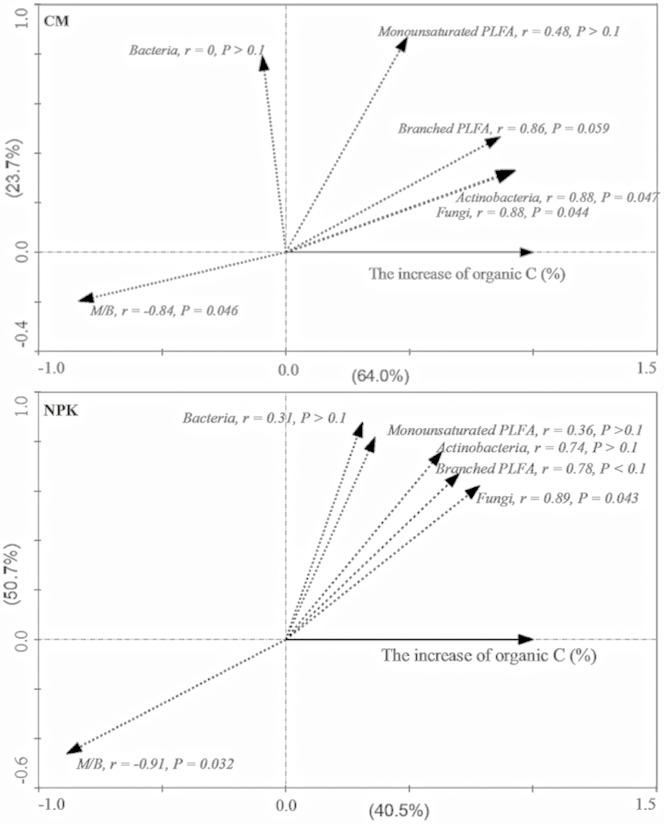
Relationship between increased organic C and the abundance of microbial PLFA (nmol g^–1^ aggregate) in soils. Redundancy analysis (RDA) and regression analyses were used to test relationships between the increased organic C concentration in the CM and NPK treatments compared with the control and the abundance of microorganisms in aggregates in the CM and NPK treatments.

Compared with the control, the increase of organic C in aggregates was found to be closely related with concentrations of branched PLFAs, fungi, and actinobacteria, as well as the ratio of M/B. The increased OC content of aggregates significantly (*P*<0.05) positively correlated with concentrations of fungal PLFAs for CM and NPK, and that of actinobacteria for CM (Fig. 5). The increase of OC was also found to be marginally (*P*<0.1) positively linked to the concentration of branched PLFAs, but significantly (*P*<0.05) and negatively correlated with the ratio of M/B in CM and NPK treatments.

## Discussion

### Distribution of Microbial PLFAs in Aggregates

Aggregates in soils provide a spatially heterogeneous micro–habitat for microorganisms characterized by different substrates and oxygen concentrations [38]. In this study, fungi and actinobacteria were mainly located in aggregates >250 μm. In contrast, although the highest abundance of bacteria was found in microaggregates, they were more even distributed across different aggregates than fungi and actinobacteria. However, bacteria in the silt and clay fractions accounted for 79–98% of total microbial PLFAs, but only 46–52% in large and small macroaggregates, indicating that bacteria were abundant across aggregate size classes in soil and dominant in the silt and clay fractions, while fungi and actinobacteria were mainly observed in macroaggregates [39], [40]. Using low–temperature scanning electron microscopy, Chenu *et al*. [41] found that actinobacteria and fungi displayed usually on the surface of aggregates greater than 10 μm in size, while bacteria displayed as individual cells or small colonies in clay pores under 2 μm. Thus, it is likely that actinobacteria and fungi were physically prevented from accessing the interior of microaggregates, silt and clay fractions where the habitats’ small spaces were limiting for fungi and actinobacteria [42]. In contrast, the protective habitats in microaggregates provided niches for bacteria through exclusion of predators (protozoa) and competition with fungi, because the predation regimen acts as a major structuring force for the bacterial community [20], [43].

Sessitsch *et al*. [20] suggested that the availability of nutrients in finer aggregates like silt and clay fractions or in smaller aggregates within macroaggregates could stimulate bacterial growth and thus bacterial diversity. Fungi and actinobacteria have mainly been found in large and small macroaggregates because they have a broader range of extracellular enzymes and prefer the particulate organic matter (POM) or the recalcitrant organic C such as lignin and hemicellulose that is rich in macroaggregates [40], [44].

Previous studies have shown that microbial biomass was lowest in the silt and clay fraction and highest in the 1,000–2,000 μm fraction [2], [45]. In our study, the concentration of total microbial PLFAs was also highest in small macroaggregates and lowest in the clay fraction, showing a decreasing pattern with reduction of aggregate size from 250–2,000 μm to <2 μm fractions (Fig. 3d). We found that 20–year compost applications significantly (*P*<0.05) increased the concentration of microbial PLFAs in microaggregates and the clay fraction compared with the control, while NPK did not. However, compost–induced stimulation did not drastically alter the distribution pattern of microbial PLFAs in soil aggregates. Previous studies demonstrated that long–term fertilization and conversion from conventional tillage to no–tillage did not affect the distribution pattern of microorganisms in aggregates of a clay loam in Sweden [20] and a subtropical paddy soil in China [22]. These results indicated that the distribution pattern of microorganisms in aggregates was mainly controlled by aggregate size and responded to a lesser extent to management practices like fertilization.

### Relationship between Microbial PLFAs and OC in Aggregates

The 20–year application of compost significantly increased organic C concentrations in all aggregates compared with the control, but fertilizer NPK exerted a much weaker effect (Table 2). The increase of organic C primarily depended on input levels of exogenous organic material and turnover of organic C [7], [46]. Our previous study showed that the decomposition rate of per unit organic C in CM–treated soil was lower than in NPK–treated soil [47]. Thus, we argued that more efficient accumulation of organic C in CM–treated soil was not only attributable to higher annual inputs of organic C as compost or crop residues, but also to lower decomposition rates of per unit organic C. In the present study, fungi and actinobacteria, which prefer particulate organic matter (POM), were reduced more efficiently in compost–treated soil, which might lead to the recalcitrant organic C accumulation in the CM treatment. However, correlation analysis showed that fungi and actinobacteria positively correlated with the increase in organic C in aggregates, possibly because they were mainly distributed in macroaggregates.

Killham *et al*. [48] and Dungait *et al*. [49] pointed out that the turnover of organic C depends on the accessibility of the OC to decomposer organisms or catalytic enzymes rather than its recalcitrance and location in the soil pore network. Organic C turnover was faster in pores with neck diameters greater than 4 μm than in those with smaller neck diameters [50]. Likewise, a previous study found the mineralization ratio of added fructose ^13^C in pores >291 μm to be 41.4% during a 13–day incubation, being significantly higher than in pores <97 μm [51]. In the present study, organic C in macroaggregates showed a more rapid rate of increase than in other aggregates in spite of the fact that macroaggregates had higher abundances of microorganisms, especially fungi and actinobacteria as discussed above. Although it is possible that more exogenous organic material was accumulated in macroaggregates [14], we found that the M/B ratio declined in parallel with an increase in aggregate size (Fig. 4), and was negatively related to the rise in organic C in aggregates (Fig. 5). Our findings suggest that organic C turnover in aggregates is also dependent on the abundance and composition of the microbial community.

Ruamps *et al*. [51] suggested that the pore–scale distribution patterns of microorganisms did not occur by chance, but were the result of interactions between microorganisms and their habitat. Blagodatsky and Smith [52] found that the aeration of intra–aggregates changed with the formation of aggregates and could be completely anoxic. The increased organic C in microaggregates (free or within macroaggregates) caused an increase in pore–filling organic matter (mainly as POM or amorphous organic materials), and in turn reduced the proportion of large pores [53], [54]. As a result, the proportion of pores <4 μm increased significantly in compost–treated soil while the effective diffusion coefficient of oxygen in both treatments significantly decreased in our study (Fig. 2). Soil aeration and pore networks are known to play a large role in the sequestration and turnover of organic C [34], and when the oxygen concentration in soil air reduced to ≤10%, the accumulation of organic C increased by slowing the oxidation of soluble forms of organic C [55], because aerobes decompose organic C more efficiently than facultative or obligate anaerobes [56]. The M/B ratio has generally been found to decrease with the development of anaerobic conditions or reduction in oxygen availability in soils [36], [57]. Monounsaturated PLFAs mainly represent G^–^ bacteria, which can utilize a variety of organic C sources and promote the decomposition of organic C under well–aerated conditions [58], [59], while branched PLFAs being dominantly composed of G^+^ bacteria [36], which possess a greater proportion of peptidoglycan, which contains significant quantities of N–acetylglucosamine that is a precursor of relatively decay–resistant organic matter [60]. Thus, we considered that the aeration status would improve as aggregate size reduced [61], which in turn would control the activity and composition of microorganisms and organic C mineralization in aggregates. The more effective accumulation of organic C in macroaggregates than in other aggregates was at least partly due to the higher concentrations of branched PLFAs and low M/B ratios. However, the organic C accumulation was least in microaggregates in NPK treatment, was mainly due to a low input of exogenous organic materials rather than the decomposition of organic C [54].

The increase in organic C in aggregates in NPK–treated soil was significantly (*P*<0.05) lower than in CM–treated soil. Yu *et al*. [54], in an incubation study using the same soils, demonstrated that compost more efficiently improved the stability of organic C, even labile organic C such as carbohydrate, in the silt plus clay fraction compared to NPK. John [62] suggested that the high turnover of organic C in the clay fraction was caused by a relatively high enrichment of organic C from fresh residues and/or microbial biomass. In NPK–treated soil, the newly increased organic C was mainly derived from root residues and exudates, while also from compost in CM–treated soil. Compost had been fermented for 2 months before use. During composting, the proportion of labile, hydrophilic, plant–derived organic compounds gradually reduced. In contrast, that of more stable hydrophobic moieties, including lignin–derived phenols and microbial–derived carbohydrates, increased [63]. When these microbial–derived carbohydrates are incorporated into the soil, they are not usually utilized by microorganisms and can become stabilized by mineral particles. It is likely that low annual input and high decomposability of exogenous organic material in NPK–treated soil resulted in low increases in organic C in the silt and clay fractions compared to CM. Furthermore, the mass proportion of macroaggregates was exponentially related to OC concentrations in the silt plus clay fractions [54]. Greater input of recalcitrant OC and reduction in monounsaturated PLFAs is likely to have been critical for the observed increase in organic C in the silt and clay fractions. Our findings suggest that organic C accumulation in aggregates and aggregation increased the proportion of pores <4 μm and reduced the effective diffusion coefficient of oxygen, gradually leading to a change in micro–habitats from those favorable for aerobes to those beneficial for facultative or obligate anaerobes in the CM–treated soil [37]; the shift in microbial community composition in turn altered organic C turnover and accumulation in aggregates.

## Conclusions

Compost application more effectively increased organic C concentrations in aggregates, and accelerated the formation of macroaggregates than fertilizer NPK. The proportion of pores <4 μm was significantly (*P*<0.05) increased in CM treatment, while the reduction effective diffusion coefficient of oxygen showed a decrease order: control>NPK>CM (*P*<0.05). The distribution pattern of microorganisms in aggregates was primarily controlled by aggregate size and responded to a lesser extent to management practices like fertilization. The concentration of actinobacteria were more significantly (*P*<0.05) reduced in all aggregates in CM–treated soil than in NPK–treated soil, whereas fungi were only reduced in macroaggregates under compost and NPK application compared with the control, resulting in recalcitrant organic C accumulated effectively in CM treatment. The M/B ratio, which was lowest in CM treatment in all aggregates, increased with the reduction of aggregate size in tested soils. Increased organic C in aggregates in CM– and NPK–treated soils was marginally (*P*<0.10) positively correlated with the concentration of branched PLFAs but significantly (*P*<0.05) negatively correlated with M/B ratios. More efficient accumulation of organic C in aggregates in CM–treated soil than in NPK–treated soil was probably due to the reduction of monounsaturated PLFAs and increase of branched PLFAs, which resulted from the change of micro–habitats in aggregates from one more favorable for aerobes to that beneficial for facultative or obligate anaerobes with the formation of macroaggregates.
